# Clevidipine for Perioperative Blood Pressure Control in Infants and Children 

**DOI:** 10.3390/ph6010070

**Published:** 2013-01-15

**Authors:** Joseph D. Tobias, David B. Tulman, Sergio D. Bergese

**Affiliations:** 1 Departments of Anesthesiology & Pain Medicine, Nationwide Children's Hospital and The Ohio State University, 700 Children's Drive Columbus, OH 43205, USA; 2 Departments of Anesthesiology and Neurological Surgery, Wexner Medical Center, The Ohio State University, 410 W 10th Ave Columbus, OH 43210, USA; E-Mail: David.Tulman@osumc.edu (D.B.T.); Sergio.Bergese@osumc.edu (S.D.B.)

**Keywords:** clevidipine, pediatric hypertension, acute hypertension

## Abstract

Various pharmacologic agents have been used for perioperative BP control in pediatric patients, including sodium nitroprusside, nitroglycerin, β-adrenergic antagonists, fenoldopam, and calcium channel antagonists. Of the calcium antagonists, the majority of the clinical experience remains with the dihydropyridine nicardipine. Clevidipine is a short-acting, intravenous calcium channel antagonist of the dihydropyridine class. It undergoes rapid metabolism by non-specific blood and tissue esterases with a half-life of less than 1 minute. As a dihydropyridine, its cellular and end-organ effects parallel those of nicardipine. The clevidipine trials in the adult population have demonstrated efficacy in rapidly controlling BP in various clinical scenarios with a favorable adverse effect profile similar to nicardipine. Data from large clinical trials regarding the safety and efficacy of clevidipine in children is lacking. This manuscript aims to review the commonly used pharmacologic agents for perioperative BP control in children, discuss the role of calcium channel antagonists such as nicardipine, and outline the preliminary data regarding clevidipine in the pediatric population.

## 1. Introduction

Various factors may result in perioperative hypertension in the pediatric-aged patient including renal failure or insufficiency, volume overload, or activation of the sympathetic nervous system [1,2,3]. Perioperative blood pressure (BP) control may be even more problematic and of greater consequence in specific clinical scenarios such as surgery for congenital heart disease (CHD) or patients with intracranial pathology where hypertension may result in excessive bleeding or disruption of suture lines. Once treatable causes of hypertension such as pain, hypercarbia, and hypoxemia are excluded, pharmacologic control of BP may be indicated. During the perioperative period, there are several options for rapid BP control in infants and children including sodium nitroprusside (SNP), nitroglycerin (NTG), labetalol, fenoldopam, and calcium channel antagonists, including nicardipine [3,4,5]. This manuscript reviews the commonly used pharmacologic agents for perioperative BP control in children, discusses the role of calcium channel antagonists such as nicardipine, and outlines the preliminary data regarding clevidipine in the pediatric population.

## 2. Agents for Blood Pressure Control

### 2.1. Sodium Nitroprusside

SNP is a direct-acting, non-selective vasodilator that dilates both resistance and capacitance vessels. Vasodilatation of resistance vessels leads to a reduction of systemic vascular resistance while capacitance vessel dilatation augments venous pooling thereby decreasing preload. SNP is administered for BP control during hypertensive urgencies and emergencies due to its rapid onset of action as well as a rapid peak hypotensive effect (within 2 minutes). These properties allow easy titration for BP control by continuous intravenous infusion. Its offset is equally as rapid as BP returns to baseline values generally within 3–5 minutes following discontinuation of the infusion. In many centers, SNP remains the time-honored therapy for the rapid control of acute hypertension in the pediatric population [6]. However, given the paucity of data regarding the use of this agent in children and the lack of safety and pharmacokinetic information, it may be that SNP popularity merely relates to introduction in 1955 when there were limited options for the pharmacologic control of BP. Despite introduction into clinical medicine in 1955, there are currently no FDA-approved indications for SNP use in the pediatric aged patient.

The cellular effects of SNP on the smooth muscle of the vasculature are due to the intracellular release of nitric oxide (NO), which activates guanylate cyclase leading to an increase in the intracellular concentration of cyclic guanosine monophosphate (cGMP). cGMP decreases the availability of intracellular calcium, resulting in a net decrease of free cytosolic calcium which leads to vascular smooth muscle relaxation. Although SNP is a frequently chosen agent for the perioperative control of BP, there remain significant concerns with its adverse effect profile in both the pediatric and the adult populations (Table 1) [7,8,9,10,11,12,13,14,15]. The potential for excessive hypotension exists even when SNP is used within currently recommended dosing guidelines. Cardiovascular collapse is another risk in the event of inadvertent overdosing. These concerns mandate intra-arterial BP monitoring. Although generally thought to be rare, a recent report documented cyanide concentrations within the toxic range in 7 of 63 (11%) pediatric patients who were treated with SNP [15].

**Table 1:** Adverse effect profile of sodium nitroprusside.

Tendency for excessive hypotension
Increased ICP in patients with altered intracranial compliance
Light sensitivity can lead to medication degradation during infusion
Increased intrapulmonary shunt
Platelet dysfunction
Activation of the sympathetic nervous system
Rebound hypertension with discontinuation of the infusion
Tachyphylaxis with prolonged use
Cyanide and thiocyanate toxicity
Cardiovascular effects with may shift myocardial oxygen delivery-demand ratio: Reflex tachycardia Increased contractility Decreased diastolic blood pressure Potential for coronary steal

### 2.2. Nitroglycerin

The other nitrovasodilator, nitroglycerin (NTG), is primarily used to treat coronary ischemia in adults. NTG is a directing acting vasodilator with a mechanism of action that is similar to SNP as its vasodilatory effects are the result of NO production. It is a short-acting agent with a rapid onset of action (1 to 2 minutes), a brief duration of action (3 to 5 minutes), and a plasma elimination half-life of 1.5 minutes. The vasodilating effects are primarily limited to the venous side (capacitance vessels) leading to a reduction of venous return with concomitant reduction in stroke volume and cardiac output.

As a direct acting vasodilator, NTG is associated with a similar adverse effect profile as SNP including the potential to increase intracranial pressure (ICP) in patients with altered intracranial compliance and inhibition of hypoxic pulmonary vasoconstriction (HPV) [16,17,18,19]. When compared with SNP, there is less of a decrease in diastolic BP and limited tachycardia, which maintains coronary perfusion pressure and time. The renin-angiotensin system is not activated and rebound hypertension does not occur. Additional adverse effects include methemoglobinemia, which is more common when the total dose exceeds 5 mg/kg, the need to use specialized tubing since NTG may bind to the plastic of standard intravenous tubing, and an increase in the bleeding time due to inhibition of platelet aggregation. The effect on platelet aggregation is less than that seen with SNP and again tends not to be clinically significant. Pediatric data regarding the safety and efficacy of NTG in the treatment of hypertension is limited. Most importantly, given its primary effect on the capacitance vessels, NTG has a limited role and limited efficacy in BP control in the pediatric population.

### 2.3. Beta-Adrenergic Antagonists

β-adrenergic antagonists may be used as the primary agent or as adjuncts to other agents for the control of BP. In many scenarios, these agents are combined with SNP and used to control the reflex tachycardia and stimulation of the sympathetic nervous system [20]. Agents in common clinical use include labetalol, esmolol, metoprolol, and propranolol. Labetalol is a competitive antagonist at α_1_, β_1_, and β_2_ adrenergic receptors. BP control results from its antagonistic effects at the α_1_ and β_1_ adrenergic receptors resulting in decreased systemic vascular response, decreased force of contractility, and a slowing of heart rate (HR). When compared with other β-adrenergic antagonists, the α adrenergic blockade of labetalol prevents the increase in systemic vascular resistance which results from unopposed β_2_-adrenergic blockade. With intravenous administration, labetolol has an onset of action is 5–10 minutes, with duration of action of 3–6 hours. The latter may be problematic if adverse effects occur.

Advantages of labetalol include limited and clinically insignificant adverse effects on ICP. When substituted for SNP, labetolol has been shown to control BP and decrease ICP [21,22]. Labetalol does not impair HPV and has a clinically insignificant effect on oxygenation [23]. When used in combination with SNP, labetalol minimizes SNP dose requirements, improves PaO_2_, and prevents rebound hypertension [24]. Adverse effects include heart block, prolonged hypotension following discontinuation of the infusion, heart failure, bronchospasm, nausea and vomiting. Labetalol also has the potential to worsen hyperkalemia, especially in children with depressed renal function.

The efficacy of labetalol in reducing MAP intraoperatively seems dependent on the anesthetic technique. Labetalol tends to be most effective with the use of inhalational anesthetic agents and less so during nitrous-narcotic or neuroleptic anesthetic techniques. Given its longer half-life, labetalol is most commonly administered as intermittent bolus doses of 0.1–0.5 mg/kg. When a more prolonged effect is required, a continuous infusion may be used. To date, there remain a limited number of reports regarding the use of a labetalol infusion to control BP in the pediatric population [25,26]. In a retrospective review of 13 children, ranging in age from 4 to 15 years, labetalol was effective in controlling hypertension of various etiologies in the pediatric ICU setting. In 12 of 15 episodes, a bolus dose was administered prior to the infusion. The bolus dose varied from 0.2 to 1 mg/kg while the infusion requirements varied from 0.25 to 1.4 mg/kg/hour (mean dose of 0.78 ± 0.39 mg/kg/hour). BP decreased from a mean of 143/99 mmHg to 116/72 mmHg while labetolol was judged to be clinically effective in all patients. In a retrospective review of antihypertensive therapy in 37 patients ≤ 24 months of age, Thomas *et al*. reported that labetalol was as effective as SNP or nicardpine in controlling BP when using the primary end point of a 20% reduction of systolic BP within 8 hours [26]. Of the 15 patients that received labetalol, the authors calculated the breakpoint for dose saturation which they reported was 0.59 mg/kg/hour although their formulary and labeling recommendations include doses up to 3 mg/kg/hour. They also noted that hypotension requiring cessation of the infusion was more common in infants with ischemic or traumatic brain injury.

In addition to labetalol, the short acting, cardioselective β_1_-adrenergic antagonist esmolol may have a role in the acute control of BP during the perioperative period and in the ICU setting. Esmolol has a rapid onset of action (3 minutes) with an elimination half-life of 9.2 minutes. Rapid metabolism due to hydrolysis by erythrocyte esterases results in an elimination half-life of approximately 9 minutes with no effect from renal or hepatic insufficiency. The shorter half-life of esmolol allows it to be titrated by intravenous infusion with tighter HR and BP control than can be achieved with intermittent bolus doses of other agents such as labetalol, propranolol or metoprolol. The short half-life allows for quick reversibility in the event of adverse effects.

As with labetalol, esmolol may be used as a primary agent or as an adjunct to control the tachycardia from direct acting vasodilators such as SNP. Edmondson *et al.* demonstrated dose-dependent reductions in SNP requirements with increasing doses of esmolol which resulted in reductions of HR, cardiac output, plasma renin activity, and catecholamine level [20]. The authors also noted improved arterial oxygenation with the decreased dose of SNP. During the perioperative period, esmolol has been used most commonly to control BP following surgery for CHD. In a phase IIIb, multicenter, double-blind, randomized, dose-ranging trial, esmolol was administered to 116 children less than 6 years of age following surgical repair aortic coarctation [27]. The patients were randomized to receive low dose (125 µg/kg/min), medium dose (250 µg/kg/min) or high dose (500 µg/kg/min) esmolol infusions with the addition of other medications if the BP was not controlled within 5 minutes. There was a significant decrease in systolic BP from baseline in all groups (mean of 9.6 mmHg) with no difference in the BP decrease or the need for additional medications between the 3 infusion ranges. No serious adverse events were reported. Similar efficacy was reported by Wiest *et al*. in their cohort of 20 patients following surgery for congenital heart disease [28]. Esmolol was titrated to achieve a BP ≤ 90th percentile for age. Although esmolol was effective in 19 of 20 patients, the authors noted higher infusion requirements than previous studies (mean of 700 µg/kg/min, range 300 to 1000 µg/kg/min). Requirements were highest in patients following coarctation repair (mean dose of 830 *versus* 570 µg/kg/min).

β-adrenergic antagonists such as esmolol reduce cardiac contractility and should not be used in patients with acute decompensated heart failure. Additional concerns include associated bradycardia and the potential to precipitate bronchospasm. Given its relative β_1_-selectively, esmolol has been shown to have limited effects on pulmonary resistance and compliance [29]. Esmolol and other β-adrenergic antagonist should not be used for BP control when hypertension results from α-adrenergic stimulation as seen with catecholamine excess related to pheochromocytoma or drug intoxications (cocaine). Additionally, in many clinical scenarios in the pediatric population, esmolol as the sole agent may not effectively control BP outside of the pediatric congenital heart surgery population and as noted, significantly higher doses than those commonly used in the adult population may be required.

### 2.4. Fenoldopam

Fenoldopam mesylate is a direct acting vasodilator, which was developed by chemical modification of the dopamine molecule. Stimulation of the post-synaptic dopamine_1_-receptor vasodilates the vascular beds of the kidney, mesentery, and skeletal muscle thereby lowering MAP. The pharmacokinetic profile is such that rapid intravenous titration can effectively control MAP. Peak effects on MAP are observed within 5–15 minutes and steady-state serum concentrations achieved within 60 minutes. Clinical effects of fenoldopam dissipate rapidly when the infusion is discontinued. Despite preliminary evidence demonstrating its efficacy in controlling MAP in various scenarios in the pediatric population, fenoldopam is not widely utilized for BP control in either the adult or pediatric populations [30,31]. One of the primary advantages of fenoldopam is that it may increase renal blood flow, urinary flow, and induce natriuresis. Although the FDA approved dose is 0.2 to 0.8 µg/kg/min due to the risk of tachycardia at higher doses, the only large, prospective pediatric trial demonstrated that doses of 0.8–1.2 µg/kg/min were required to significantly lower BP [32]. Fenoldopam is also significantly more costly than other agents, including SNP [33]. Currently, its primary clinical use is to augment urine output and improve fluid balance in various fluid overload states; usually in the ICU setting [34].

### 2.5. Calcium Channel Antagonists (Nicardipine)

Given concerns with the available agents, the search continues for the optimal pharmacologic agent for BP control. Calcium channel antagonists impede the movement of calcium through voltage-gated calcium channels in cardiac muscle and the smooth muscle of the vascular, reducing the force of contraction and vasodilatation. Based on chemical structure, the calcium channel antagonists can be separated into subgroups of phenylalkylamines, benzothiazepines, and dihydropyridines. In addition to slight differences in the chemical structures among these subgroups, there is also variation in regards to their effects on the inotropic, chronotropic, and dromotropic function of the myocardium and their effects on the smooth muscle of the vasculature. Phenylalkylamine calcium channel antagonists such as verapamil are relatively selective for the myocardium. They have minimal vasodilatory effects compared with dihydropyridines and are used mostly in the treatment of ischemia related to coronary artery disease or to control atrial arrhythmias. Benzothiazepines such as diltiazem are an intermediate class between the phenylalkylamines and dihydropyridines in their selectivity for the vascular calcium channels. Although occasionally used for BP control, their most common clinical indication is the treatment or prevention of arrhythmias. Dihydropyridine calcium channel antagonists such as nicardipine and amlodipine remain the most commonly used agents of this group for BP control. Their primary physiologic effect is on the smooth muscle on the vasculature resulting in vasodilatation with effects primarily on the arterial side thereby limiting decreases in preload.

When considering the calcium channel antagonists, nicardipine remains the most commonly used agent of this class for BP control in both the adult and pediatric population [35]. When administered intravenously, nicardipine has a rapid onset of action of 1–2 minutes, with an elimination half-life of approximately 40–60 minutes. Metabolism is primarily hepatic without the production of active metabolites. Its primary physiologic effect is vasodilation with limited effects on chronotropy, dromotropy, and inotropy. In isolated tissue preparations, nicardipine has been shown to have at least twice the selectivity for vascular *versus* cardiac smooth muscle when compared to other calcium channel antagonists (verapamil, diltiazem, and nifedipine) [36]. The relative vascular selectivity of nicardipine is further supported by clinical trials demonstrating either no change or an improvement in cardiac output following its administration [37,38]. Given these properties, nicardipine has seen widespread use in both the adult and pediatric population for BP control, including the perioperative period [39,40,41,42,43]. In general, the literature has demonstrated the efficacy of nicardipine in controlling BP with a favorable adverse effect profile including limited tachycardia when compared to other direct acting vasodilators including SNP. One consideration with nicardipine use is the longer elimination half-life thereby resulting in a longer duration of effect following discontinuation of the infusion [41,44,45].

## 3. Clevidipine

Clevidipine (Cleviprex^®^, The Medicines Company, Parsippany, NJ, USA) is a short-acting, intravenous calcium channel antagonist of the dihydropyridine class. It undergoes rapid metabolism by non-specific blood and tissue esterases with a half-life of less than 1 minute [46]. Because of the esterase metabolism, no dosing adjustments are required for renally or hepatically impaired patients. It is currently approved for use in adults by the Food & Drug Administration for BP reduction when oral therapy is not feasible or desirable. As a dihydropyridine, its cellular and end-organ effects parallel those of nicardipine. The clevidipine trials in the adult population have demonstrated its efficacy in rapidly controlling BP in various clinical scenarios with a favorable adverse effect profile similar to nicardipine. Despite these reported advantages, clevidipine may be more costly when compared to other agents [47].

One of the initial clinical trials compared clevidipine to placebo in adult cardiac patients presenting with preoperative hypertension defined as a systolic BP ≥ 140 mmHg [48]. A clevidipine infusion ranging from 0.4 µg/kg/min up to a maximum of 8 µg/kg/min effectively reduced systolic BP by ≥ 15% in 92.5% of patients compared to 17.3% of placebo patients (failure rate of 82.7% in placebo-treated patients). The target BP was achieved in a median time of 6 minutes (95% confidence interval: 6 to 8 minutes) after initiation of the infusion. HR increased from a mean of 71 to a maximum of 84 beats per minute (BPM). There were no differences between clevidipine and placebo in regards to the adverse effect profile.

A subsequent trial (ESCAPE-2), also in adult cardiac surgical patients, compared clevidipine with placebo in the treatment of postoperative hypertension in adult cardiac surgical patients [49]. Again, hypertension was defined as a systolic BP ≥ 140 mmHg with the goal being BP reduction of ≥ 15% from baseline. Patients were randomized to receive either placebo or clevidipine (0.4 up to 8 µg/kg/min). The target BP was achieved in 91.8% of the patients receiving clevidipine *versus* 20.4% of those treated with placebo (*p* < 0.0001). The median time to achieve BP control was 5.3 minutes (95% confidence interval: 4 to 7 minutes). No statistically significant change in HR occurred and no significant adverse effects were noted.

When compared with other antihypertensive agents in the adult population, preliminary data also support the efficacy of clevidipine. When compared with SNP, nitroglycerin or nicardipine for the treatment of acute hypertension in adult cardiac surgery patients, BP control was more effective with clevidipine when compared with nitroglycerin (*p* = 0.0006) or SNP (*p* = 0.003) [50]. No difference was noted when comparing clevidipine with nicardipine with a target systolic BP range of 75–145 mmHg. However, when considering the more narrow target range of 105-145 mmHg, clevidipine was more effective for BP control compared to nicardipine (*p* = 0.0231). Based on these data, the authors noted the potential for clevidipine to provide tighter BP control [50]. Also of note, mortality was lower in patients receiving clevidipine than in patients receiving SNP (*p* = 0.04).

To date, there have been a limited number of reports regarding the use of clevidipine in the pediatric population, including two isolated case reports and a retrospective case series comprising a total of 10 patients (Table 2) [51,52,53]. Overall these reports have demonstrated effective BP control with clevidipine in clinical scenarios including intraoperative administration during resection of a pheochromocytoma. In addition, two larger retrospective series provide additional information regarding the use of clevidipine in the pediatric population [54,55].

The first retrospective report included 14 pediatric patients undergoing surgery for CHD [54]. The patients ranged in age from 11 months to 15 years (7.2 ± 4.6 years) and in weight from 5 to 45 kg (24.1 ± 13.1 kgs). Clevidipine was administered as a continuous infusion to control postoperative BP in 6 patients, as a continuous infusion for intraoperative control of MAP during cooling and cardiopulmonary bypass (CPB) in three patients, or as a bolus dose for the treatment of hypertension during emergence from anesthesia in five patients. In the six patients who required clevidipine for postoperative BP control, three of which followed repair of an aortic coarctation, the infusion was started at 1 μg/kg/min and increased in increments of 0.5–1 μg/kg/min every 2–3 minutes as needed. For all six patients, the dosing requirements varied from 1 to 7 μg/kg/min (2.0 ± 1.2 μg/kg/min) to successfully reach the target BP within 5 minutes. The duration of the postoperative infusion varied from 8 to 19 hours. Either intravenous or oral propranolol was administered to two patients to treat a HR increase ≥ 20 beats/min. Both were receiving clevidipine at the higher end of the dosing range (5–7 μg/kg/min). In the six patients who received clevidipine for BP control following surgery, four were transitioned to oral therapy (propranolol or captopril) while the infusion was weaned and discontinued in the other two without need for further therapy. In three patients in the cohort, clevidipine was administered intraoperatively as a continuous infusion to control MAP during cooling (core body temperature of 28–32 °C) while on CPB. The target MAP could not be achieved in any of these patients despite clevidipine doses up to 10 µg/kg/min. Effective BP control was eventually achieved with either SNP or phentolamine. In the remaining five patients, intermittent bolus dosing of clevidipine was used to control BP during emergence from anesthesia prior to tracheal extubation in the operating room for fast-track anesthesia. Nine bolus doses (10–15 µg/kg/dose) were administered to these five patients and resulted in a decrease of the MAP from 97 ± 6 mmHg to 71 ± 5 mmHg (*p* < 0.01). Following the bolus dosing, HR increased by 10 ± 4 beats/minute (p < 0.01). In one patient, the HR increase was ≥ 20 beats/minute, but no treatment was necessary. Other than this mild increase in HR, no other adverse effects were observed during clevidipine administration. Overall, the authors noted that clevidipine was effective in controlling elevations of BP following surgery for CHD and during emergence from anesthesia. However, it proved ineffective in controlling MAP during cooling while on CPB. The failure of clevidipine may relate to the inactivation of calcium channels during this degree of hypothermia. The same mechanisms do not appear to pertain to other pharmacologic agents, such as phentolamine and SNP, whose actions are dependent on other receptor systems as both were effective in controlling the MAP during hypothermia.

A second retrospective study evaluated the efficacy of clevidipine in providing controlled hypotension to limit intraoperative blood loss during spinal surgery in a cohort of 20 adolescents, ranging in age from 14–18 years and in weight from 46–96 kg [55]. Intraoperative anesthesia included a total intravenous anesthetic technique utilizing propofol, remifentanil, and dexmedetomidine. To maintain the MAP at 55–65 mm Hg, clevidipine was administered starting at 0.5–1 μg/kg/min and then increased in increments of 0.5 to 1 μg/kg/min every 2–3 minutes to achieve the desired MAP. The time to achieve the target MAP was ≤ 5 minutes in 15 of the 20 patients and 5–10 minutes in the other 5 patients. The maintenance infusion rate of clevidipine varied from 1 to 5 μg/kg/min (2.9 ± 0.7 μg/kg/min). A mild HR increase was noted with the administration of clevidipine (baseline of 76 ± 14 to 92 ± 11 beats/min, p < 0.05). The HR increase was ≥ 20 beats/minute in four patients and intermittent doses of metoprolol were administered to three patients. Excessive hypotension was not noted. When the clevidipine infusion was discontinued, MAP returned to baseline within 5 minutes in 16 of the 20 patients and within 10 minutes in the other four patients.

**Table 2:** Anecdotal reports of clevidipine use in the pediatric population.

Authors and Reference	Patients Demographics	Clevidipine Dosing and Outcome
Tobias, J.D.* et al.* [51]	16-year-old, 57 kg adolescent with renal failure and hypertension presented for anesthetic care during urgent placement of a peritoneal dialysis catheter. BUN was 53 mg/dL with a serum creatinine of 7.2 mg/dL.	BP was 162/108 mmHg on arrival to the preoperative area. After arrival in the OR, a clevidipine infusion was started at 1 μg/kg/min. Within 3-4 minutes, the BP was 117/66 with no change in HR. After anesthetic induction, the BP was 124/79 mmHg and the clevidipine infusion was decreased to 0.5 μg/kg/min and maintained at that dose throughout the procedure. During emergence, the infusion was increased to 1 μg/kg/min. The infusion was increased to 2 μg/kg/min on arrival to the PACU to control an increased BP to 144/105 mmHg. After administration of his usual morning dose of amlodipine, the clevidipine infusion weaned off.
Towe, E.* et al.* [52]	Retrospective review in a cohort 10 pediatric-aged patients, ranging in age from 9 to 18 years. Indications for clevidipine included control of perioperative hypertension (4), intraoperative controlled hypotension (5), and to improve distal perfusion during a toe-to-finger implant (1).	The clevidipine infusion was started at 0.5 to 1 μg/kg per minute and titrated up to 3.5 μg/kg per minute as needed. Higher doses were required for controlled hypotension than for other indications. Clevidipine was used preoperatively (1), intraoperatively (2), postoperatively. Intermittent doses of metoprolol were required to control reflex tachycardia in 2 of the 10 patients.
Bettesworth, J.G.* et al.* [53]	15-year-old, 60-kg adolescent with a pheochromocytoma for elective resection after preoperative preparation with phenoxybenzamine.	Following anesthetic induction and before surgical incision, a clevidipine infusion was started at 0.5 μg/kg/min. During surgery, the clevidipine was titrated from 0.5 to 3 μg/kg/min to maintain a MAP within 20% of the patient's baseline level. Esmolol was administered to control mild tachycardia (HR 110 to 135 beats/min). After the adrenalectomy, there was no abrupt change in the hemodynamic parameters. The clevidipine infusion was continued at 0.5 to 1 μg/kg/min during the end of the case and to the PACU. A single dose of labetalol (10 mg) was administered and the clevidipine infusion was discontinued.

BP = blood pressure; BUN = blood urea nitrogen; MAP = mean arterial pressure; OR = operating room; PACU = post-anesthesia care unit; HR = heart rate; PICU = pediatric intensive care unit.

## 4. Conclusions

As clevidipine shares many of the beneficial physiologic effects of nicardipine in addition to having a much more predictable and shorter duration of action, dosing may be advantageous in situations requiring the rapid and tight control of BP. In particular, the rapid resolution of its effects following discontinuation of the infusion may be beneficial in situations where excessive hypotension may be deleterious. A summarized assessment comparing clevidpine to the other available antihypertensive agents is outlined in Table 3. Preliminary data in the adult population has demonstrated potential advantages of clevidipine over SNP and nitroglycerin. Although limited, data from the pediatric population indicate that the agent may have a role for BP reduction in the pediatric population in various scenarios including following surgery for CHD, as an agent for intraoperative controlled hypotension, and in the perioperative control of hypertension of several etiologies including renal failure. However, a large, prospective clinical trial has not been performed, to date, to demonstrate its efficacy.

**Table 3:** Physiologic and pharmacologic attributes of antihypertensive agents.

Agent	Rapid Onset	Rapid Offset	Easily Titrated	Risk of Tachycardia	Adverse ICP Effects	Major Limitations
Sodium nitroprusside	++	++	+	+	+	Excessive hypotension.
						Risk of cardiovascular collapse.
						Cyanide and thiocyanate toxicity.
Nitroglycerin	++	++	+	-	+	Excessive hypotension.
						Primary action on capacitance vessels only.
						Binds to plastic intravenous tubing.
Labetalol	+	-	-	-	-	Longer duration of action.
						Excessive hypotension.
						Limited pediatric data.
Esmolol	+	+	+	-	-	Reduced cardiac contractility.
						Bradycardia
						Limited use outside of congenital heart surgery.
Fenoldopam	+	+	+	+	Unknown	Limited efficacy.
						Limited pediatric data.
Nicardipine	++	-	+	-	+/-	Longer duration of action with prolonged offset time.
Clevidipine	++	++	+	-/+	Unknown	Limited pediatric data.
						Lipid emulsion as diluent with risk of elevated triglycerides,
						contraindicated with soy or egg allergy, and risk for
						bacterial contamination of vials.

Clevidipine is commercially available in a concentration of 0.5 mg/mL in 50 or 100 mL vials. Because of solubility issues, it is provided in a lipid solution and is contraindicated in patients with allergy to eggs, egg products, soy beans or soy products as well as disorders of lipid metabolism. Although no meaningful alteration of serum triglyceride levels has been noted in adult patients with clevidipine administration [56], data from the pediatric population demonstrates the potential for a mild elevation of triglyceride levels when clevidipine is administered with propofol. In the study of Towe *et al*., the triglyceride level was elevated (328 mg/dL, normal values 50–150 mg/dL) in one patient following the prolonged (4–6 hour) administration of propofol at doses of 50–100 µg/kg/min and clevidipine at 2–3.5 µg/kg/min (both medications are delivered in a similar lipid diluents) [52]. In addition to these issues, the phospholipids of the solution may support bacterial growth. Clevidipine now contains edetate disodium 0.005%, which inhibits bacterial growth for up to 12 hours. Vials should be used within 12 hours of puncture, and strict aseptic technique is required.

To date, the youngest patient to receive clevidipine in published reports has been 11 months of age [54]. Although nicardipine has been used safely in the neonatal population and has been shown to have limited effects on myocardial contractility, given the catastrophic effects of verapamil in neonates and infants, caution is suggested when using clevidipine in neonates and young infants until additional data is available regarding its effects on myocardial contractility [55,57]. Given its efficacy in the adult cardiac population, its pharmacokinetic profile, rapid elimination, and the preliminary successes in the pediatric-aged patient, future trials in various clinical scenarios in the pediatric population are warranted.
